# RAPIDIRON Trial follow-up study — the RAPIDIRON-KIDS Study: protocol of a prospective observational follow-up study

**DOI:** 10.1186/s13063-023-07740-z

**Published:** 2023-12-20

**Authors:** Richard J. Derman, Roopa B. Bellad, Mrutyunjaya B. Bellad, Jesse Bradford-Rogers, Michael K. Georgieff, Zubair H. Aghai, Simal Thind, Michael Auerbach, Rupsa Boelig, Benjamin E. Leiby, Vanessa Short, S. Yogeshkumar, Umesh S. Charantimath, Manjunath S. Somannavar, Ashalata A. Mallapur, Ramesh Pol, Umesh Ramadurg, Radha Sangavi, Basavaraj V. Peerapur, Nasima Banu, Praveen S. Patil, Amaresh P. Patil, Subarna Roy, Phaniraj Vastrad, Dennis Wallace, Hemang Shah, Shivaprasad S. Goudar

**Affiliations:** 1 Thomas Jefferson University (TJU), Philadelphia, USA; 2 KLE Academy of Higher Education and Research (KAHER), Jawaharlal Nehru Medical College (JNMC), Belagavi, India; 3 University of Minnesota, Minneapolis, USA; 4 Nemours Children’s Health, Philadelphia, USA; 5 Georgetown University School of Medicine, Washington, DC, USA; 6 S. Nijalingappa Medical College (SNMC), Bagalkot, India; 7 Raichur Institute of Medical Sciences (RIMS), Raichur, India; 8 Model Rural Health Research Unit (MRHRU), Sirwar, India; 9 The Children’s Investment Fund Foundation (CIFF), New Delhi, India

**Keywords:** Anemia, Iron deficiency anemia, Neurodevelopment, Anemia in pregnancy

## Abstract

**Background:**

Anemia is a worldwide problem with iron deficiency being the most common cause. When anemia occurs in pregnancy, it increases the risk of adverse maternal, fetal, and postnatal outcomes. It induces preterm births and low birth weight (LBW) deliveries, long-term neurodevelopmental sequelae, and an increased risk of earlier onset of postnatal iron deficiency. Anemia rates are among the highest in South Asia, and India’s National Family Health Survey (NFHS-5) for 2019–2021 indicated that over half of pregnant women, and more than 65% of children, in the country are classified as anemic (Sciences IIfP, National Family Health Survey-5, 2019–21, India Fact Sheet). In 2021, the parent *RAPIDIRON Trial* (Derman et al., Trials 22:649, 2021) was initiated in two states in India, with the goal of assessing whether a dose of intravenous (IV) iron given to anemic women during early pregnancy results in a greater proportion of participants with normal hemoglobin concentrations in the third trimester and a lower proportion of participants with LBW deliveries compared to oral iron. As a follow-up to the *RAPIDIRON Trial,* the *RAPIDIRON-KIDS Study* will follow the offspring of previously randomized mothers to assess, neurobehavioral, hematological, and health outcomes.

**Methods:**

This prospective observational cohort study will follow a subset of participants previously randomized as part of the *RAPIDIRON Trial* and their newborns. Study visits occur at birth, 6 weeks, 4 months, 12 months, 24 months, and 36 months and include blood sample collection with both maternal and infant participants and specific neurobehavioral assessments conducted with the infants (depending on the study visit). The primary outcomes of interest are (1) infant iron status as indicated by both hemoglobin and ferritin (a) at birth and (b) at 4 months of age and (2) the developmental quotient (DQ) for the cognitive domain of the Bayley Scales of Infant Development Version IV (BSID-IV) at 24 months of age.

**Discussion:**

This *RAPIDIRON-KIDS Study* builds upon its parent *RAPIDIRON Trial* by following a subset of the previously randomized participants and their offspring through the first 3 years of life to assess neurodevelopmental and neurobehavioral (infants, children), hematological, and health outcomes.

**Trial registration:**

ClinicalTrials.gov NCT05504863, Registered on 17 August 2022. Clinical Trials Registry – India CTRI/2022/05/042933. Registered on 31 May 2022.

## Administrative information

**Table:** 

Title {1}	RAPIDIRON Trial follow-up study — the RAPIDIRON-KIDS Study: protocol of an observational follow-up study
Trial registration {2a and 2b}	Clinicaltrials.gov: NCT05504863, https://clinicaltrials.gov/ct2/show/NCT05504863; Clinical Trials Registry – India: CTRI/2022/05/042933, https://ctri.nic.in/Clinicaltrials/pmaindet2.php?trialid=68455
Protocol version {3}	V2.5 February 14, 2023
Funding {4}	This research is supported by a grant from the Children’s Investment Fund Foundation (CIFF)—R-2111–07169
Author details {5a}	Thomas Jefferson University (TJU), KLE Academy of Higher Education and Research (KAHER), Jawaharlal Nehru Medical College (JNMC), S. Nijalingappa Medical College (SNMC), Raichur Institute of Medical Sciences (RIMS), Model Rural Health Research Unit (MRHRU), Georgetown University School of Medicine, University of Minnesota, and the Children’s Investment Fund Foundation (CIFF)
Name and contact information for the trial sponsor {5b}	The sponsor for this trial is the Children’s Investment Fund Foundation (CIFF), 7 Clifford Street, London, W1S 2FT, United Kingdom
Role of sponsor {5c}	The study funder and sponsor will provide ongoing oversight of the trial by way of monthly update meetings, review of quarterly monitoring reports from the study team and providing an independent team to routinely audit the quality and safety of study practices. They will be provided deidentified data and reports for review and provide feedback but they will not have ultimate authority over study related activities.

## Introduction

### Background and rationale {6a}

Anemia is a global problem, with 2019 estimates suggesting it affected a quarter of the world’s population. These estimates further specify that 40% of children aged 6–59 months, 37% of pregnant women, and 30% of women aged 15–49 years were affected by anemia, globally, in 2019 [1]. The major cause of anemia is iron deficiency, the most common micronutrient deficiency in the world [2]. Rates of anemia and iron deficiency vary greatly by geography, population, and socioeconomic status, with markedly higher prevalence rates in low- and middle-income countries (LMICs) [1, 3].

While both the World Health Organization (WHO) and the United Nations (UN) have set global priorities and targets for reducing anemia in women of reproductive age, progress is slow and not currently on track to accomplish these goals [1]. In response, many countries — including India, Uganda, Sierra Leone, Oman, Nepal, and the Philippines — have developed national anemia strategies, with varying success. Countries that have successfully reduced anemia prevalence tend to have more multisectoral, comprehensive strategies involving a variety of national and international actors [1].

South Asia is home to some of the highest rates of anemia globally, with the most recent National Family Health Survey (NFHS)-5 conducted in 2019–2021, indicating that greater than half of all pregnant women in India are anemic [4]. Additionally, iron deficiency anemia (IDA) is a major contributing factor to the high rate (> 65%) of children (6–59 months of age) in India who are classified as anemic (defined as a hemoglobin level of < 11 g/dL) [4].

IDA during pregnancy poses risks to not only the pregnant individual, but also the future offspring. Optimal fetal, neonatal, and childhood brain growth and development require adequate iron [3]; however, women with moderate to severe anemia during the late second and early third trimesters of pregnancy are often unable to make up their iron deficit solely with the use of oral iron. Thus, despite active transport via the placenta, insufficient iron may be transmitted to the developing fetus with consequent negative sequelae including long-term neurodevelopmental impairment of the offspring [3, 5].

A recent study in China found that while oral iron during pregnancy may improve maternal iron status, 45% of women still deliver iron-deficient offspring, suggesting that oral iron may not function optimally for the developing fetus [6]. Published evidence confirms that iron deficiency in infancy is associated with statistically significant decreases in cognitive and behavioral functioning which may persist for decades despite iron repletion [7]. The presence of long-term neurodevelopmental effects, even when iron deficiency is diagnosed and promptly treated in infancy, implies that the prevention of neonatal and postnatal iron deficiency through optimal fetal iron loading during pregnancy is the preferred course of action [3].

While there are studies of intravenous iron to treat anemia during pregnancy currently ongoing in multiple countries (Malawi [8], Nigeria [9]), none, to our knowledge, are assessing long-term neurodevelopmental impacts in the offspring.

#### Parent trial

In 2021, *Reducing Anemia in Pregnancy in India: the RAPIDIRON Trial* [10] was initiated in two states in India with the goal of assessing if a single dose of intravenous (IV) iron (either ferric carboxymaltose or ferric derisomaltose) given to women with moderate anemia during pregnancy will result in a greater proportion of participants achieving normal hemoglobin concentrations in the third trimester and/or a lower proportion of participants delivering a low birth weight (LBW) baby compared to a control group given oral iron as standard of care. The two IV iron formulations selected for the parent *RAPIDIRON Trial* enhance the overall generalizability of study results associated with the use of IV iron. The parent trial also compares the two IV iron formulations with the aim of demonstrating the effectiveness of either in the treatment of IDA when compared to oral iron. Both IV formulations selected for use in the *RAPIDIRON Trial* are approved and available commercially in India; they allow single-dose infusions of up to one gram of iron; they have a proven track record of efficacy and availability in many countries of the world; they are associated with low rates of adverse events; high-quality studies show no difference in severe side effects among available IV iron formulations; and there is greater probability that inclusion of more than one single-dose formulation will be instrumental in driving down market prices and perhaps lead to public sector pricing and greater utilization. Oral iron is being used as the comparator arm as this is currently the first-line treatment for both mild and moderate IDA in pregnancy.

#### Current study

The *RAPIDIRON-KIDS Study* extends the parent *RAPIDIRON Trial* by following a subset of the previously randomized mothers as well as their offspring after birth to assess neurodevelopmental, hematological, and health outcomes. The overarching objective is to determine whether offspring born to *RAPIDIRON Trial* mothers who received IV iron, compared with oral iron, will be born with better iron stores and achieve superior neurodevelopment and growth at specific time points during the first three years of life.

#### Rationale for study inference strategy

The parent *RAPIDIRON Trial* was designed to compare the effects of each of the two IV iron treatments to the effects of oral iron on pregnant women [10]. In designing the *RAPIDIRON-KIDS* follow-up study and as a logical extension of the presently ongoing *RAPIDIRON* randomized clinical trial (RCT), two alternative inferential approaches were considered — a three-arm comparison using multiple hypothesis tests, comparable to the approach used in the parent RCT, and a single comparison of the average treatment effect of the two IV arms to the effect on the oral arm. Taking into consideration the absence of any prior evidence of a differential impact of the two IV treatments, the resource requirements and participant burden of the neurodevelopmental follow-up studies required for the *RAPIDIRON-KIDS Study*, and the sample size requirements necessary to conduct a well-powered study, the approach using the comparison of the average treatment effect of the two IV arms to the effect on the oral arm was selected as the most feasible approach for conducting this follow-up study.

### Objectives {7}

#### Specific aims

The specific aims of *RAPIDIRON-KIDS* are:

- To provide evidence that a single dose of IV iron (either ferric carboxymaltose or ferric derisomaltose) given to pregnant women in the early second trimester of pregnancy during the *RAPIDIRON Trial* will prove more effective for prevention of neonatal and postnatal iron deficiency in the offspring than oral iron given to pregnant women per the parent trial protocol;
- To assess whether the offspring of women randomized in the parent trial and provided IV iron have better neurodevelopmental outcomes compared to the offspring of women treated with oral iron; and
- To determine longer-term hematologic effects in previously randomized mothers through assessment of hematologic values and a documented history of transfusion and hospitalization. The study will also assess quality of life based on the use of a validated instrument.

#### Primary hypotheses

- Infants born to *RAPIDIRON Trial* mothers in the IV iron arms will have higher hemoglobin concentrations and serum ferritin concentrations (a) at birth (determined by cord blood) and (b) at four months of age compared to infants born to mothers in the oral iron arm; and
- Offspring born to *RAPDIRION Trial* mothers given IV iron will have higher developmental quotients (DQs) on the cognitive domain of the Bayley Scales of Infant Development, Version 4 (BSID-IV) at two years of age compared to offspring born to *RAPIDIRON Trial* mothers given oral iron treatment as recommended by the World Health Organization (WHO)

### Trial design {8}

This is a prospective observational cohort study of a subset of participants (and their offspring) previously randomized as part of the *RAPIDIRON Trial.* Data collection will be conducted at six time points: birth/delivery, six weeks, four months, 12 months, 24 months, and 36 months postpartum.

#### Parent study

The *RAPIDIRON-KIDS Study* recruits a subset of women solely from the *RAPIDIRON Trial* and extends the maternal follow-up period, allowing for follow-up of participants and their offspring. The *RAPIDIRON Trial* is a three-arm randomized-controlled trial assessing the use of a single-dose IV iron formulation — either ferric carboxymaltose in intervention arm #1 or ferric derisomaltose in intervention arm #2 — compared to oral iron as standard of care among pregnant women in India with IDA. The *RAPIDIRON Trial* hypothesizes that: (1) a greater proportion of participants in the IV iron arms will achieve normal hemoglobin concentrations at either of two time points in the late third trimester, compared to those in the oral iron arm; and (2) a lower proportion of participants in the IV iron arms will deliver low birth weight infants compared to those assigned to the oral iron arm.

The *RAPIDIRON Trial* was initiated in March 2021 with randomization now complete. Further information about the *RAPIDIRON Trial* including intervention and control arm details, randomization and consent processes, ethical approvals, statistical analyses, and data management can be found in the published protocol paper [10].

## Methods: participants, interventions, and outcomes

### Study setting {9}

The study will be conducted in India with participants recruited during the *RAPIDIRON Trial* pre-delivery visits at participating primary and community health centers (PHCs and CHCs) from three districts in the state of Karnataka. Post-delivery study visits will occur at specified hospitals in the research areas — KLES Dr. Prabhakar Kore Charity Hospital and Medical Research Centre in Belagavi, S.N. Medical College in Bagalkot, and Raichur Institute of Medical Sciences in Raichur. The parent study was conducted across two states in India (Karnataka and Rajasthan); due to resource considerations, this follow-up study will only be conducted in Karnataka state.

### Eligibility criteria {10}

Study participants will be mother-infant dyads consisting of women, residing in Karnataka state, who were randomized and treated for IDA during the *RAPIDIRON Trial* and their newborns. Additional eligibility criteria will include:

#### Inclusion criteria

- Informed consent of the *RAPIDIRON Trial* participant for their study inclusion and that of their offspring for ongoing participation in the *RAPIDIRON-KIDS Study*
- Expressed intent to remain in the designated Karnataka research area for delivery and during the follow-up period (3 years) of the *RAPIDIRON-KIDS Study*
- For the offspring — live-born singleton infants of maternal participants from three sites in Karnataka randomized and treated in the *RAPIDIRON Trial*, with informed consent provided by the mother

#### Exclusion criteria

- Unwillingness of maternal participant to provide *RAPIDIRON-KIDS Study* consent for their self and offspring

### Who will take informed consent? {26a}

Initial screening for interest and eligibility for the *RAPIDIRON-KIDS Study* will occur starting at the sixth *RAPIDIRON Trial* visit (26–30 weeks of pregnancy). This visit occurs at the participant’s PHC/CHC site and involves monitoring of pregnancy, drawing blood samples for iron status and other analysis, and collecting other data specific to the parent *RAPIDIRON Trial*. In addition, study staff will provide current *RAPIDIRON Trial* participants with information about the *RAPIDIRON-KIDS Study* and an opportunity to consent. Potential participants are encouraged to take home this information and to share and discuss with trusted family and friends before making a decision regarding participation. Potential participants will be able to provide consent any time between the sixth *RAPIDIRON Trial* visit (26–30 weeks of pregnancy) up until delivery, as long as they are not yet in active labor.

A trained healthcare provider or research staff member at the PHC/CHC will obtain informed consent, ensuring each potential participant and/or immediate family member has adequate time to read the consent form and ask questions. As literacy levels will vary and may present challenges, the consent process will include a verbal review of the consent form. If the potential participant cannot read, it will be read aloud by a person unaffiliated with the study. Alternatively, a Study Coordinator or a designee may read the consent, but in the presence of a witness who is unaffiliated with the study. Potential participants will be given an opportunity to discuss the procedures and ask questions. Fair balance will be maintained while describing the risks and benefits of study participation and no undue pressure will be placed on the potential participant to enroll. It will be explained that the lack of participation will not affect the usual and anticipated standard of care, nor their current participation in the parent *RAPIDIRON Trial*.

Following review of the consent form and study procedures, the potential participants will be asked to sign the form if they are willing to participate in the study. If the participant is unable to sign, they will be asked to use their thumbprint to indicate written approval. In both cases, the unaffiliated person will also sign the consent form. Both the research staff and the study participant will retain signed copies of the form.

### Additional consent provisions for collection and use of participant data and biological specimens {26b}

Potential participants will be providing consent for both themselves and their future offspring to participate in this follow-up study. All staff responsible for obtaining consent will be trained and certified in the protection of human subjects as well as study-specific consent procedures. The research team has developed a model consent form that will be provided to all sites.

An eligible individual may refuse to participate at the time of recruitment or consent and may choose to withdraw from the study at any time after enrollment.

During the informed consent process, participants will be provided information regarding the biological specimens to be collected as well as the timing and reasons for collection. They will also be informed of confidentiality processes — that their data will be deidentified and stored in a secure database, and no identifiable data will ever be released. When providing consent, participants will also be asked to consent to share deidentified data with other researchers.

## Interventions

### Explanation for the choice of comparators {6b}

This follow-up study will involve no interventions beyond those implemented in the parent study; the interventions are described in detail in Derman et al. [10] and summarized in the introduction of this manuscript.

### Intervention description {11a}

This follow-up study will involve no interventions beyond those implemented in the parent study; the interventions are described in detail in Derman et al. [10] and summarized in the introduction of this manuscript.

### Criteria for discontinuing or modifying allocated interventions {11b}

When participants are entered into the study, delivery of the IV iron for the parent study will have been completed. This study has no criteria for discontinuing or modifying allocated interventions.

### Strategies to improve adherence to interventions {11c}

Because the interventions from the parent study are completed prior to enrollment in this follow-up study, we are not monitoring adherence as a part of this study. However, data on adherence will be monitored in the parent study as detailed in Derman et al. [10], and the data on adherence from that study will be available for use in the subsequent analyses of data collected for this follow-up study.

### Relevant concomitant care permitted or prohibited during the trial {11d}

At each study visit, study staff will ask the participant about any unscheduled hospitalizations that may have occurred since their last visit. This information will be collected on a specific unscheduled hospitalization form. Health care providers seeing enrolled participants will make decisions about care that may be required due to pregnancy or delivery-related complications or other health-related issues. It is expected that the provider will offer usual standard of care to the participants in treating non-anemia-related issues or other non-pregnancy/delivery-related health concerns that may arise.

### Provisions for post-trial care {30}

The last scheduled study visit will occur at approximately three years post-delivery. It would be expected that healthcare providers will deliver usual standard of care throughout the study and indicate the need for specific follow-up care for both mother and baby.

### Outcomes {12}

#### Primary outcomes

The first of two primary outcomes of this study addresses offspring iron status as indicated by hemoglobin and ferritin concentrations (a) at birth and (b) at four months of age. Hemoglobin concentration is used to assess the presence of anemia and to calculate iron in the red cell mass. Serum ferritin concentration primarily assesses iron stores and is important for calculating total body iron along with other parameters [3]. The sample obtained at birth — using cord blood — directly assesses fetal loading, which is associated with the use of maternal iron therapy.

Measuring iron status at four months allows for the assessment of whether greater fetal iron loading results in longer-lasting postnatal improvements in hematologic and iron status during a period of rapid neurodevelopment. The World Health Organization (WHO) recommends exclusive breastfeeding with no supplemental foods until an infant reaches six months of age [11]. However, infants born at term who have delayed cord clamping, who are breastfed and not supplemented with iron, will be depleted of fetal iron stores at about six months of age, with potential consequent impact on brain development [12]. Additionally, oral iron supplementation beginning at four months of age to address or attempt to prevent iron deficiency may result in a shift toward a more pathogenic microbiome. Babies are born relatively immunocompromised and such a shift can place them at greater risk for diarrheal illness earlier in life when they are less able to tolerate infection [13]. If the associated hypothesis is true, babies born to mothers in the IV iron arms will not have a need for postnatal oral iron therapy as early in life as the infants whose mothers received prenatal oral iron. The infants with mothers who received IV iron are expected to be replete with prenatal IV iron, as evidenced by being born with greater total body iron, as indexed by hemoglobin and ferritin concentrations, as well as greater storage iron, as indexed by ferritin concentration, at birth and four months of age.

The second primary outcome relates to differences in the DQ of the cognitive domain of the BSID-IV [14] at 24 months of age. The BSID is a validated tool for assessing general developmental abilities and is routinely used in large-scale trials, including in India [15, 16], to detect deficits in motor, language, social-emotional, and cognitive domains. Maternal iron deficiency anemia has been shown to reduce offspring BSID scores (normed and referred to as developmental quotients (DQs)) starting at one year and beyond [17, 18].

#### Secondary outcomes

This study will evaluate a variety of secondary outcomes in the offspring participants starting at delivery and four months of age. Maternal iron deficiency during pregnancy has been associated with a variety of adverse neurodevelopmental outcomes in the offspring including reduced hippocampal capacity, behavioral problems, and autism [3]. From a neurodevelopmental perspective, four months is the first age at which reliable assessments of the integrity of the cortex are possible [19]. Cortical maturation is critical for motor, cognitive, and social well-being [20]. Prior to four months, infants’ behaviors are primarily subcortical and reflexic (as characterized by the presence of primitive reflexes) [21]. At four months, cortical control is evidenced by controlled motor movements, social smiling, and novel visual object discrimination memory [22]. Based on the *RAPIDIRON* hypotheses, infants loaded with prenatal IV iron will be neurologically more mature with faster speed of processing and better discrimination memory performance at four months than infants whose mothers received prenatal oral iron.

A secondary hypothesis of this study is that infants born to women randomized to the IV iron arms of the parent *RAPIDIRON Trial* will have superior neurobehavioral development compared to infants born to women randomized to the oral iron arm. To test this secondary hypothesis, the following outcomes will be assessed in the offspring:

- Performance on Preferential Looking Time test, a behavioral test that assesses recognition learning and memory [23]. This test assesses the integrity of the hippocampus brain region and will be applied when the child is four and 12 months of age. The test leverages the brain development principle of novelty preference. The hippocampus is rapidly developing in the last trimester through the first 18 postnatal months. Iron deficiency reduces hippocampal capacity [3]. Infants are presented with an object (or picture of an object) and are expected to familiarize themselves with it. After a time delay, the familiar object is presented along with a novel object. The infant is expected to spend more time (> 60% of total looking time) observing the novel object.
- Performance on Ages & Stages Questionnaire-3 (ASQ-3), a questionnaire designed to obtain parental input regarding a child’s general developmental milestones which can be used for children from one month to five and a half years of age [24]. Mothers will be expected to provide answers for the age-appropriate questionnaire that will be used when the child is 12 months of age.
- Scoring of performance on motor, language, and social-emotional domains of BSID-IV at 24 months.
- Performance on Behavioral Rating Scale, which was devised by the pioneer iron researcher Betsy Lozoff [25]. It is coded from a video recording of a child undergoing the BSID and it assesses engagement, tractability, exploration, hesitancy, and activity level. All domains that are dopamine-dependent and reported by Lozoff and others as perturbed by iron deficiency effects on dopamine metabolism will be administered during visits to a subset of enrolled children (50 per arm) at 24 months of age.
- Internalizing and externalizing behaviors on the Child Behavior Checklist for Ages 1.5 to 5 years (CBCL/1.5–5), which is a survey where parents rate items on a three-point Likert scale. A recent study reported that children with iron deficiency at birth had higher internalizing behavior problem scores at five years compared to those born iron sufficient [26]. This will be conducted at 36 months of age.
- Risk of autism diagnosis via the India Scale for Assessment of Autism (ISAA) [27], a test similar to the standard screening tool for autism (the Modified Checklist for Autism in Toddlers) that has been validated, and was developed specifically, for the Indian population [28]. Iron deficiency during early pregnancy is associated with a higher risk of autism in the offspring [3] and will be assessed at 36 months of age.

Another secondary hypothesis of this study is that children born to women randomized to the two IV iron arms of the parent *RAPIDIRON Trial* will have better iron stores at 12 months compared to children born to women randomized to the oral iron arm. To evaluate this hypothesis, a number of standard hematologic and iron indices will be assessed in all subjects. The trajectory of differences in iron status in children will be graphically depicted from birth through 12 months. Tracking key indicators of infant development and iron status through 12 months of age will provide additional critical data regarding associations of early iron deficiency with autism and enable a better understanding of growth and hemoglobin trajectories related to overall neurocognitive development.

The parent *RAPIDIRON Trial* hypothesizes that IV iron treatment early in pregnancy will reduce iron-deficiency-associated LBW, primarily due to premature delivery or intrauterine growth restriction [10]. Both are risks for low iron stores at birth and poor postnatal growth in the offspring [12]. It is hypothesized that offspring born to mothers in the oral iron group, compared to the IV iron groups, are more likely to be iron deficient during their fetal life, at birth, and during infancy. Prolonged iron deficiency can negatively impact linear growth, and chronic iron deficiency during the rapid phase of growth in infancy and early childhood results in vulnerability for growth impairment [13]. Iron plays an important role in tissue oxygen delivery and transport of electrons within cells as well as acting as a co-factor for essential enzymatic reactions. IDA likely delays growth by creating a hypoxemic condition and inhibiting insulin-like growth factor-I (IGF-1) [29, 30]. Hypoxemia due to iron deficiency anemia can also lead to an increase in hypoxia-induced growth factor (HIF-1α), which in utero has been shown to decrease human growth hormone mRNA levels [31]. In infants and young children with iron deficiency anemia, iron therapy increased serum IGF-1 levels and growth velocity [32]. Several other studies have reported lower body weight, height, head circumference, and growth velocity in anemic children and improvement in growth parameters with iron therapy [33–35]. To assess growth and nutrition in this study, we will measure the weight, length (height), and head circumference of the offspring at each study visit and plot them on the WHO standard pediatric growth curves [36]. From these data, weight for age, length for age, and head circumference for age *Z*-scores will be generated and growth velocities will be calculated. Additionally, we will administer the Infant and Child Feeding Index (ICFI), a composite index used to measure feeding practices, which has been previously used in the Indian context and allocates scores based upon breastfeeding, dietary diversity, meal frequency, etc. [37]. Data regarding iron-supplementation and iron-fortified foods will also be collected.

In addition to assessing secondary outcomes in the offspring, this study also enables continued contact with a sample of *RAPIDIRON* maternal participants. The interaction with these mothers during this follow-up study is an important means of appreciating the longer-term effects of the treatments used during the parent trial. Maternal secondary outcomes to be explored include the following: iron status at four, 12, and 24 months post-delivery; breastfeeding practices; life quality, as measured by the World Health Organization Disability Assessment Schedule 2.0 (WHODAS 2.0) [38]; and rates of hospitalization and transfusion through three years post-delivery.

### Participant timeline {13}

The participant timeline is shown in Table 1.

**Table 1:**
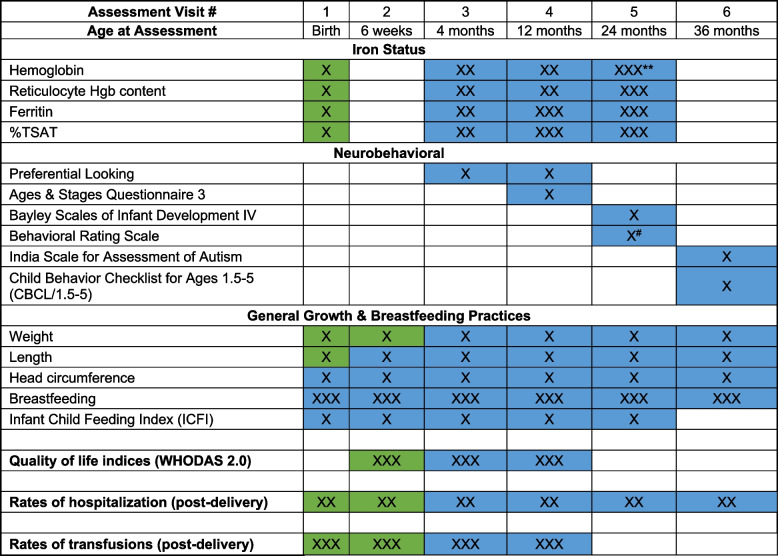
Schedule of RAPIDIRON-KIDS Study

If a participating mother experiences an intercurrent pregnancy within 36 months post-delivery, a blood sample will be collected and analyzed to obtain a complete set of iron indices

^*^The RAPIDIRON study will provide much of the information for the first two visits (as made clear by the different color boxes and keys). Cord blood is the source of iron status for Visit 1

^**^If no difference in iron status between *RAPIDIRON* treatment arms is detected at 12 months (once 12-month sample analysis is complete), 24-month maternal blood samples will not be collected

^#^Behavior Rating scale will be performed on a subset of 50 infants per arm

### Sample size {14}

While the parent *RAPIDIRON Trial* compares outcomes across all three of its randomization and treatment arms, this study was designed to compare the impact of IV iron (averaged across the two IV treatment groups) with oral iron as there is no evidence that the IV formulations will differ with respect to their effect on the primary outcomes.

Current *RAPIDIRON Trial* participants will be asked to consent to this follow-up study prior to delivery with the expectation that some will experience a stillbirth or neonatal death and, thus, not provide valuable information on the infants. In addition, cord blood data will not be obtainable or analyzable for a certain percentage of live births. We have accounted for the non-availability of these samples in the statistical analysis plan. Thus, we plan on consenting approximately 525 *RAPIDIRON Trial* participants (~ 175 per parent trial arm).

Each primary hypothesis will be tested at the 0.05 level. A total of four hypothesis tests are proposed for hypothesis one. First, two tests will be conducted on data collected at birth, with separate tests comparing offspring born to mothers across both IV iron arms to those born to mothers in the oral arm with respect to hemoglobin level and log-transformed ferritin levels at the alpha = 0.025 level. In developing the testing strategy, we assumed that treatment effects on infant iron status at four months are biologically plausible only if the treatment affected iron status at birth. Consequently, the comparable test will be conducted at four months post-delivery only if those tests show differences at birth. In all cases, the null hypothesis is that the mean hemoglobin or ferritin levels across the IV arms are the same as that in the oral iron arm. Limited comparisons of infant hemoglobin levels in south Asia and Africa suggest that the differences in both hemoglobin levels and log ferritin levels in infants born to mothers with and without IDA differ by at least 0.8 standard deviations. We want to assure that this study has sufficient power to detect an effect in the range of 0.3 to 0.4 standard deviations. We anticipate that approximately 85% of the enrolled sample (147 infants per arm) will provide data for our primary iron outcomes at birth. When evaluated in the context of the proposed mixed effects model where we assume an intraclass correlation coefficient of 0.5, a sample size of 294 IV iron vs. 147 oral gives more than 80% power to detect a difference in means at birth of 0.31 standard deviations at the alpha = 0.025 level. Since demonstrating the effects at four months requires that the hypothesis tests at birth reject the null hypothesis, the overall study power to detect a difference of 0.3 standard deviations or more at four months is greater than 80%.

The primary outcome for hypothesis two is the BSID DQ measured at two years of age. The average score across the two IV iron arms will be compared with the oral arm at the alpha = 0.05 level. We expect to obtain data for approximately 85% of consented participants at two years for a sample of at least 147 per arm. Effect sizes in the range of 5 to 7.5 are generally clinically meaningful for the BSID. This sample size gives more than 90% power to detect a difference in means of five units on the BSID mean score assuming a standard deviation of 15 at the alpha = 0.05 level.

### Recruitment {15}

In this follow-up study, potential participants will be recruited during parent *RAPIDIRON Trial* visits prior to birth, as early as the sixth *RAPIDIRON Trial* visit (at 26–30 weeks of pregnancy). All potential participants previously consented to randomization, treatment, and participation in the parent *RAPIDIRON Trial* (see protocol paper for additional details) [10]. Current *RAPIDIRON Trial* participants at the three participating sites in Karnataka will be given information about this follow-up study, encouraged to ask questions of the research team, and asked whether they are interested in participating and providing consent for themselves and their future offspring. They will also be able to take home information and consent materials to review with their husbands and/or other trusted family and friends before consenting to participate. Participants may provide consent up until delivery, as long as they are not yet in active labor.

## Assignment of interventions: allocation

### Sequence generation {16a}

N/A — as this is an observational follow-up study, there is no randomization. Details of the parent trial randomization scheme are discussed in the *RAPIDIRON Trial* protocol paper [10].

### Concealment mechanism {16b}

N/A — as this is an observational follow-up study, there is no randomization. Details of the parent trial randomization scheme are discussed in the *RAPIDIRON Trial* protocol paper [10].

### Implementation {16c}

N/A — as this is an observational follow-up study, there is no randomization. Details of the parent trial randomization scheme are discussed in the *RAPIDIRON Trial* protocol paper [10].

## Assignment of interventions: blinding

### Who will be blinded {17a}

Research staff conducting the neurodevelopmental assessments and laboratory analysis will be blinded to the participants’ treatment assignment in the parent study. Details regarding the parent study intervention and blinding are provided in the *RAPIDIRON Trial* protocol paper [10].

### Procedure for unblinding if needed {17b}

As stated above, individuals conducting the neurodevelopmental assessments and laboratory analysis will be blinded to the participants’ treatment assignment in the parent study. Details regarding the parent study intervention and blinding are provided in the *RAPIDIRON Trial* protocol paper [10].

## Data collection and management

### Plans for assessment and collection of outcomes {18a}

The parent *RAPIDIRON Trial* will be the source of most data for the first two visits, whereas the last four visits will involve assessments done specifically for the *RAPIDIRON-KIDS Study.* Please see Table 1 for the schedule of visits and an overview of the overlap of assessments with the parent trial. Initial screening and consent for this follow-up study will occur during parent *RAPIDIRON Trial* visits, prior to birth.

#### Iron status

Offspring iron status will be measured at birth using cord blood, collected as part of the parent trial protocol, to assess fetal iron loading. Infant hemoglobin and iron indices will be determined via venipuncture at four months and by a heel stick at 12 months. Maternal iron status will be measured via blood draws conducted at the four-, 12-, and 24-month visits. However, if no difference in maternal iron status between *RAPIDIRON Trial* treatment arms is detected at 12 months (once 12-month sample analysis is complete), 24-month maternal blood samples will not be collected.

#### Neurodevelopmental assessments (offspring)

A number of general and targeted assessment tools will be used to test neurodevelopment-related outcomes and associations. These tools were chosen based on prior published studies related to iron status, cultural competence, previous experience in conducting them in India, and familiarity of the research team (to better enable training and study conduct).

The BSID-IV will be administered [14] at 24 months to measure the neurodevelopment-related primary outcome as well as to provide information for multiple secondary outcomes. In addition, administration of the BSID-IV at 24 months will be video recorded and coded according to a Behavioral Rating Scale which assesses domains specifically affected by iron deficiency [25, 39]. The Preferential Looking Time test will be applied at four months and 12 months to assess recognition learning and memory [23]. Parental input on the offspring’s general developmental milestones will be gathered via the ASQ-3 at 12 months [24]. The CBCL/1.5–5 will be administered at 36 months along with the ISAA.

#### Growth and nutrition (offspring)

To assess growth trajectories, head circumference, length (height), and weight of offspring will be measured at each of the six time points: birth, six weeks, four months, 12 months, 24 months, and 36 months. The ICFI will also be administered at each time point to gather information regarding nutritional intake.

#### Maternal secondary outcomes

In addition to collecting information regarding offspring, we will utilize surveys to gather data related to the maternal participant. This includes the WHODAS 2.0 to measure quality of life and the recording of rates of transfusions at four months and 12 months and of rates of hospitalizations (maternal or offspring) at each time point.

### Plans to promote participant retention and complete follow-up {18b}

In order to promote retention and completion of study visits, this study utilizes Accredited Social Health Activists (ASHAs) already present in the communities of participating CHCs and PHCs. ASHAs, with authorization and support from supervising health officials, will support the study team by accompanying participants to study visits, as they do in the parent *RAPIDIRON Trial*. ASHAs who have received sensitization for *RAPIDIRON* and *RAPIDIRON-KIDS* can also serve as an additional source of information about this study. If a participant withdraws from the study or is lost to follow-up, information will be recorded on specific study data collection forms.

### Data management {19}

This study will utilize JNMC’s existing Data Management System (DMS) developed for the *RAPIDIRON Trial* and expanded for use with *RAPIDIRON-KIDS*. Data are initially collected on paper-based forms and then transferred to regional data centers for data entry into a centralized Research Electronic Data Capture (REDCap) database [10]. Participants will maintain the same ID numbers provided to them in the *RAPIDIRON Trial.* Data validation, confidentiality, and blood sample collection practices are further detailed in the *RAPIDIRON Trial* protocol [10].

### Confidentiality {27}

This follow-up study will utilize the same participant identification codes (IDs) used in the parent *RAPIDIRON Trial*. At the time of enrollment in this follow-up study, participants will complete consent forms and contact information/locator forms which link their personal information to their IDs. These paper forms will be kept in securely locked file cabinets, accessible only by authorized staff, at each research site. After enrollment, data collected will be linked only to the participants’ ID and not their names.

### Plans for collection, laboratory evaluation, and storage of biological specimens for genetic or molecular analysis in this trial/future use {33}

Cord blood collection at birth is described in the *RAPIDIRON Trial* protocol paper [10]. Specific to this follow-up study, blood samples will additionally be collected from the maternal participant at four-, 12-, and 24-month (if applicable) study visits, and from the offspring via venipuncture at four months and heel stick at the 12-month study visit. Samples will be collected at the study hospital in the participant’s area (KLES Dr. Prabhakar Kore Charity Hospital and Medical Research Centre in Belagavi, S.N. Medical College in Bagalkot, and Raichur Institute of Medical Sciences in Raichur).

Samples will be collected by nurses at the study hospital in the respective research area—KLES Dr. Prabhakar Kore Charity Hospital and Medical Research Centre in Belagavi, S.N. Medical College in Bagalkot, and Raichur Institute of Medical Sciences in Raichur. Samples will be stored upright at 2 to 8 degrees immediately after collection and will be transferred to the central lab — KLE Hi-Tech Clinical Lab in Belagavi — within six hours. While in transport, specimens will be stored in an insulated chiller box with ice packs and a thermometer used to record the temperature during transportation. Upon arrival at the central lab, staff will complete the Laboratory Requisition Form (LRF) for each specimen before processing.

The lab will employ daily controls to ensure quality control and validation of sample analysis — these processes are further outlined in the *RAPIDIRON Trial* protocol paper [10].

## Statistical methods

### Statistical methods for primary and secondary outcomes {20a}

#### Primary data analysis

Hemoglobin and log-transformed ferritin will be modeled separately using mixed effects linear regression. Both outcomes will be measured at birth and four months, and hemoglobin will also be measured at 12 months. The mixed effects models will treat time as a categorical value and include fixed effects for time, treatment, and time by treatment interaction. Correlation among repeated measurements for each subject will be modeled through structuring the variance–covariance matrix of the residuals. Including the time by treatment interaction will allow for the estimation of mean iron indices at each measurement time for each group. Within these models, the primary hypothesis tests will be performed by comparing the average across the two IV iron groups to the oral iron group with respect to the mean values at birth and at four months using a significance level of 0.025.

Linear regression will be used for modeling the BSID DQ cognitive domain in participating children at two years of age. The primary test will be performed by comparing the average across the two IV arms to the oral iron arm at two years using a significance level of 0.05.

#### Secondary analyses

Secondary analyses will examine differences between the average of the IV arms and the oral arm on each of the secondary outcome measures. The secondary outcomes are continuous, and most are collected longitudinally. Outcomes measured at only one time point will be analyzed using linear regression. Analyses for longitudinally measured outcomes will use linear mixed models to describe how the trajectory for each outcome differs as a function of treatment arm and will include fixed effects of treatment, time, and the treatment by time interaction, with infants as a random effect. Analogous models will be generated for the secondary analysis of maternal iron levels with women as a random effect. Model results will be used to generate point and interval estimates of mean levels of the outcome measures at each developmental time for the oral arm and the average of the IV arms. Exploratory analyses will be performed to separately compare each IV arm to the oral arm and the IV arms to each other. All hypothesis tests for secondary outcomes are considered to be descriptive with p-values used as indicators of the strength of evidence of treatment differences with no plans for formal statistical inference for the secondary analyses. As such, no control for multiple comparisons is planned.

Additional descriptive analyses will examine the relationship between each of the neurodevelopmental outcomes and growth parameters and infant delivery of total body iron. These analyses will utilize linear models or linear mixed models that evaluate the regression relationship between baseline iron levels and outcome measures, controlling for the treatment arm. These models will also evaluate whether the relationship between baseline iron and neurodevelopmental outcomes is mediated by specific post-delivery nutritional measures.

### Interim analyses {21b}

An analysis of maternal hematologic indices will be completed when all women have passed the 12-month collection period. This analysis will include all available maternal blood data through 12 months. Based on the results of this analysis, the research team may decide to suspend the collection of maternal blood at 24 months. Another extensive interim analysis is not planned.

### Methods for additional analyses (e.g., subgroup analyses) {20b}

The intent-to-treat (ITT) cohort includes all study participants (mothers or infants, depending on the particular analysis) using group assignments for the mother as randomized. Analyses that examine outcomes as a function of delivery iron status will include enrolled maternal participants with sufficient delivery data to determine the iron status of the infant at delivery. Participants will be assigned to the various cohorts based on a masked data review prior to the initiation of analysis.

### Methods in analysis to handle protocol non-adherence and any statistical methods to handle missing data {20c}

The primary analysis will be performed on the intent-to-treat cohort. Additional analyses including only participants where the mother received the assigned treatment may be performed. Missing data will not be imputed.

### Plans to give access to the full protocol, participant-level data and statistical code {31c}

The trial registration details in the Clinical Trial Registry of India (CTRI) outline the plan for data sharing. Data related to the results of specific publications from this trial will be shared after de-identification. The study protocol, statistical analysis plan, and informed consent forms will also be shared.

The Trial Steering Committee will review and approve proposals from other researchers to use study data. Following approval, data necessary to achieve the aims in the proposal will be made available. Researchers interested in proposing the use of study data must communicate with representatives of the Trial Steering Committee via email — Dr. Richard Derman (Richard. Derman@jefferson.edu) and Dr. M. B. Bellad (mbbellad@hotmail.com). Data will be made available starting 3 months and ending 5 years after article publication.

## Oversight and monitoring

### Composition of the coordinating center and trial steering committee {5d}

The Trial Steering Committee is comprised of the study PI — Dr. Richard Derman — and co-investigators from the Belagavi site — Dr. Shivaprasad Goudar, Dr. Mrutyunjaya Bellad, and Dr. Roopa Bellad. The collaborative research groups at Belagavi and TJU will establish a senior leadership team that will meet on a bi-weekly basis. While the PI is ultimately responsible for trial governance and study oversight, this Steering Committee will be responsible for general oversight.

JNMC houses the trial coordinating center which is responsible for coordinating day-to-day activities at the sites in India, including initial and ongoing training, management of study staff, and laboratory coordination.

Additionally, the parent *RAPIDIRON Trial* received expert input from a multi-disciplinary Technical Advisory Group (TAG) — all of who have agreed to continue their participation in support of *RAPIDIRON-KIDS.*

### Composition of the data monitoring committee, its role and reporting structure {21a}

Independent data and safety monitoring for this follow-up study will be provided by the same Data and Safety Monitoring Board (DSMB) that oversees the primary randomized controlled *RAPIDIRON Trial* [10]. Five individuals — a Chair and four supporting members — comprise the DSMB, all of whom are free of conflicts of interest and able to unbiasedly perform their duties.

The DSMB will be responsible for reviewing all serious adverse events (SAEs) and unanticipated problems occurring in mothers and infants to determine if they pose an ongoing risk; assuring that all study activities are conducted so as to minimize risk and promote safety; assessing data quality and participant retention; and preparing reports outlining their recommendations and providing them to the study team. After data from the parent RCT have been collected for outcomes through 42 days post-delivery (which constitutes completion of the clinical component of the *RAPIDIRON Trial*), the DSMB will continue to meet approximately annually to review the data from this follow-up study. The DSMB may revise the annual meeting schedule if the workload justifies.

### Adverse event reporting and harms {22}

For this study, a SAE involves an enrolled maternal participant OR their offspring. To qualify as an SAE, at least one of the following criteria must apply to the event: (1) results in a maternal or pediatric death; (2) is life-threatening; (3) requires an unanticipated hospitalization or prolongs an existing hospitalization; (4) results in persistent or significant disability or incapacity; or (5) represents other serious or unexpected adverse events that a study investigator feels should be reported. Specific forms will be utilized to report an SAE and must be sent to the DSMB within 7 days of the notification of the event. At each scheduled DSMB meeting after recruitment, SAE data and a report summarizing recruitment and participant status will be shared for review and discussion.

### Frequency and plans for auditing trial conduct {23}

Statisticians and the PI will meet on a monthly basis to review and evaluate the quality and safety of the data. A monitoring report with deidentified data will be provided to the funder on a quarterly basis. Additionally, the independent DSMB will meet approximately annually during this trial.

### Plans for communicating important protocol amendments to relevant parties (e.g. trial participants, ethical committees) {25}

The senior leadership and research team will meet on a biweekly basis to review study progress and any potential issues. Protocol amendments, when necessary, will be reviewed by all relevant ethics committees and IRBs before implementation. If a protocol amendment will change the consent process, it will be communicated to participants in a timely and appropriate manner. The research team will take into consideration safety concerns and advice from the DSMB when choosing methods to communicate changes to study participants.

### Dissemination plans {31a}

Study results will be disseminated through a variety of means including meetings and publications. The research team will disseminate results to participants according to the guidelines of CTRI.

## Discussion

Despite ongoing national efforts to reduce the prevalence of IDA in the country, iron deficiency has become one of the top ten causes of death and disability in India for all ages and sexes (combined) in the past decade [40]. Reducing anemia during pregnancy and lactation are key targets of India’s national anemia strategy [41], as IDA during pregnancy is associated with increased risks to the mother’s health, adverse pregnancy outcomes such as low birth weight and prematurity, as well as short- and long-term deleterious effects in the offspring [3]. The ongoing *RAPIDIRON Trial* aims to improve IDA treatment during pregnancy by testing the effectiveness of a single dose of IV iron — either ferric carboxymaltose or ferric derisomaltose—compared to oral iron in achieving a non-anemic state in women having moderate anemia and reducing low birth weight deliveries. This *RAPIDIRON-KIDS Study* builds upon its parent *RAPIDIRON Trial* by following a subset of the previously randomized participants and their offspring through the first 3 years of life to assess neurodevelopmental, hematologic, and health outcomes.

This study is strengthened by being a follow-up to an ongoing RCT — therefore streamlining data collection and making it more consistent. Additionally, measurement of potential confounders, such as postnatal iron supplementation and other infant dietary practices, will help facilitate confounder control in conducting the analyses. One of the design features of this cohort follow-up study is that, for the primary analysis, we assumed that the effect of IV iron on infant outcomes could reasonably be assessed using the average effect of the two IV Iron preparations as the available literature provides no evidence to the contrary. If the two formulations do in fact have a differential effect on infant outcomes, the study has limited power to detect the effects of the individual formulations, which may be an important limitation of the study.

Assuming that the primary hypotheses of both this follow-up study and the parent *RAPIDIRON Trial* prove true without raising safety concerns and the parent trial presents evidence of economic feasibility of single-dose IV iron for treatment of IDA in pregnancy, the researchers involved in these studies intend to aggressively disseminate the research results to increase the probability that the Government of India’s Ministry of Health and Family Welfare will endorse broader and appropriate scale-up of a single-dose IV iron infusion as frontline treatment for IDA in pregnancy for women with moderate IDA. Further, dissemination of positive findings for these related studies can be globally promoted as supportive of goals to improve care and outcomes in women during pregnancy and the postpartum period and reduce the rates of anemia, iron deficiency, poor growth, and the occurrence of developmental disabilities in children.

By globally disseminating the results of these studies, we hope to impact not only India’s anemia guidelines, but also other LMICs struggling to reduce their burden of anemia. Taking a multisectoral approach, as recommended by the WHO [1], disseminating results to communities, health providers, health systems, and governmental bodies will help strengthen the case for implementation at all levels. In addition to health outcomes previously discussed, anemia is associated with fatigue and reduced productivity which, in turn, is associated with decreased income that can have far-ranging consequences to the individual, family, community, and to overall economic development [42]. The potential downstream effects of maternal anemia on offspring — e.g., increased mental health burden, reduced IQ, educational attainment, and job potential — are considerable, and strengthening the evidence base for effective treatment has the potential for great societal and economic benefit.

## Conclusion

Together, the parent *RAPIDIRON Trial* and the *RAPIDIRON-KIDS Study* aim to increase the likelihood that India’s anemia guidelines and the recommendations of respected health organizations will continue to undergo refinement. We anticipate that new and improved anemia and iron deficiency treatment approaches that are convenient, cost-effective, and feasible can be more rapidly accepted, implemented, and adequately scaled up to meet a great and urgent need.

## Trial status

Protocol version 2.5, February 14, 2023. Recruitment occurred from October 2022 to May 2023. Follow-up study visits are ongoing.

## Data Availability

The trial registration details in CTRI outline the plan for data sharing. Data related to the results of specific publications from this trial will be shared after de-identification. The study protocol, statistical analysis plan, and informed consent forms will also be shared. The Trial Steering Committee will review and approve proposals from other researchers to use study data. Following approval, data necessary to achieve the aims in the proposal will be made available. Researchers interested in proposing the use of study data must communicate with representatives of the Trial Steering Committee via email — Dr. Richard Derman (Richard. Derman@jefferson.edu) and Dr. M. B. Bellad (mbbellad@hotmail.com). Data will be made available starting 3 months and ending 5 years after article publication.

## Abbreviations

ASHA: Accredited Social Health Activist
ASQ-3: Ages and Stages Questionnaire – 3rd edition
BSID: Bayley Scales of Infant Development
CBCL/1.5–5: Child Behavior Checklist for Ages 1.5–5
CHC: Community health center
CIFF: Children’s Investment Fund Foundation
CTRI: Clinical Trials Registry of India
DMS: Data Management System
DQ: Developmental quotient
DSMB: Data and Safety Monitoring Board
ICFI: Infant and Child Feeding Index
ID: Identification
IDA: Iron deficiency anemia
IGF-1: Insulin-like growth factor I
ISAA: Indian Scale for Assessment of Autism
ITT: Intent-to-treat
IV: Intravenous
JNMC: Jawaharlal Nehru Medical College
KAHER: KLE Academy of Higher Education and Research
KLES: Karnatak Lingayat Education Society
LBW: Low birth weight
LRF: Laboratory Requisition Form
NFHS: National Family Health Survey
PHC: Primary health center
PI: Principal Investigator
RCT: Randomized controlled trial
REDCap: Research Electronic Data Capture
SAE: Serious adverse event
TAG: Technical Advisory Group
TJU: Thomas Jefferson University
WHO: World Health Organization
WHODAS: World Health Organization Disability Assessment Schedule
